# Follicular carcinoma originating from struma ovarii: A case report

**DOI:** 10.1097/MD.0000000000032658

**Published:** 2023-01-06

**Authors:** Leyi Shou, Jianfeng Lu, Junjie Yang, Huabing Wang, Lijun Sun, Hao Dong, Yiqian Jiang

**Affiliations:** a Department of Pathology, Xiaoshan Affiliated Hospital of Wenzhou Medical University, Hagnzhou, Zhejiang; b Department of gynecology, Xiaoshan Affiliated Hospital of Wenzhou Medical University, Hangzhou, Zhejiang, China; c Department of Radiology, Xiaoshan Affiliated Hospital of Wenzhou Medical University, Hangzhou, Zhejiang, China; d Department of Radiotherapy, Xiaoshan Affiliated Hospital of Wenzhou Medical University, Hangzhou, Zhejiang, China.

**Keywords:** clinical manifestations, follicular carcinoma originating from struma ovarii, pathological characteristics, treatment and prognosis

## Abstract

Follicular carcinoma originating from struma ovarii is a clinically rare low-grade malignant tumor. The pathological diagnosis of ovarian thyroid follicular carcinoma is predominantly based on the infiltrative growth and vascular involvement of tumor cell nests of different sizes in the ovarian parenchyma.

**Patient concerns:**

Here we present a case of this malignancy in which the bilateral ovaries, right oviduct wall, myometrial surface, omentum, and bladder reflex were extensively involved Microscopically, the thyroid follicles in this case showed infiltrative growth of nodules of different sizes in the ovarian stroma.

**Diagnosis:**

The epithelial layer of the follicles was atypical, but with no nuclear features of papillary thyroid carcinoma such as nuclear groove and nuclear pseudoinclusions. Immunohistochemistry showed positive expression of thyroglobulin, thyroid transcription factor-1, and cytokeratin19, with a Ki-67 index of 5% +. Immunohistochemical results combined with microscopic morphology allowed a diagnosis of follicular carcinoma originating from struma ovarii.

**Interventions:**

After exclusion of contraindications to surgery, the patient underwent surgical exploration on July 26, 2022, during which frozen pathological examination was performed.

**Outcomes:**

The patient recovered well and was discharged. At the first follow-up visit in October 2022, the patient had an excellent survival.

**Conclusion:**

The analysis of the microscopic morphological characteristics and immunohistochemistry deepened our understanding of the pathological characteristics of ovarian and thyroid follicular carcinoma, and further provides a diagnostic reference for other clinicians who will encounter these conditions in the future.

## 1. Introduction

Follicular carcinoma originating from struma ovarii is a low-grade malignant tumor, which is rare in clinical practice.^[1,2]^ Some well differentiated thyroid tumors and follicular carcinoma show small tumor cell heterogeneity, and their main diagnostic basis is their invasive growth mode and vascular involvement. Some scholars have previously referred to this well differentiated tumor as “malignant struma ovarii.”^[3]^ Roth et al believed that the above diagnosis did not clearly describe the histological type of the tumor, therefore, some ovarian stromal tumors with morphology similar to thyroid follicular carcinoma are referred to as well-differentiated follicular carcinoma of ovarian origin.^[4]^ In our case, the bilateral ovaries, right oviduct wall, myometrial surface, omentum, and bladder reflex were extensively involved. We report this case to deepen our understanding of the clinical manifestations, pathological features, treatment, and prognosis of struma ovarii follicular carcinoma.

## 2. Case presentation

**Figure 1.**
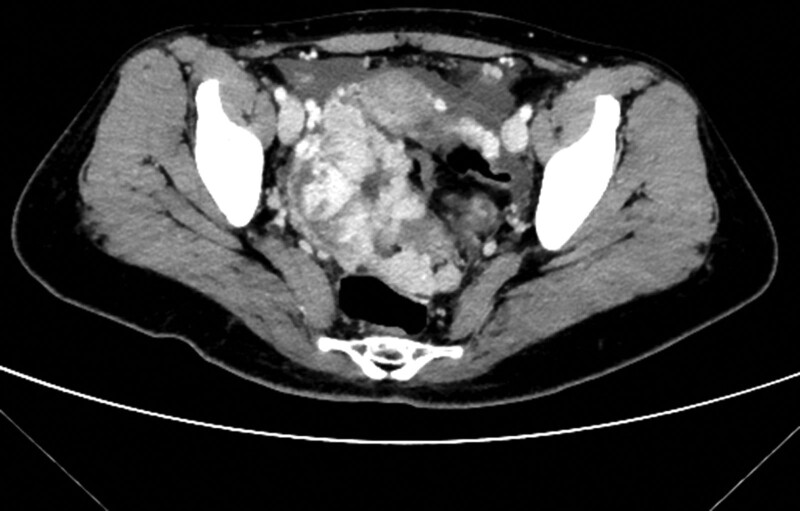
Pelvic CT showing implant metastases. CT = computed tomography.

A 37-years-old female patient was admitted to the Xiaoshan Affiliated Hospital of Wenzhou Medical University in July 2022 for lower abdominal pain that had appeared 1 month prior. Physical examination revealed coffee spots in the abdomen and limbs of the patient, and nodule like protrusions under the skin of the limbs, particularly in the left lower limb. The clinical diagnosis was neurofibroma type I, for which he had a family history. The patient was positive for the blood tumor marker CA125 51.30 ku/L. B-ultrasound gynecological examination showed that the outline of double-layer side ovary was clear, the cystic dark area was seen (size 8.5 × 5.6 × 4.4 cm^3^) shown as a strong uneven echo light mass with unclear boundary, and the internal fluid was clear, close to the right ovary. Close to the left ovary, several large lesions were observed as an uneven echo light mass sized about 4.5 × 4.0 × 2.7 cm^3^, diagnosed as “pelvic mass.” Multiple solid and cystic masses were diagnosed by pelvic computed tomography, and solid masses in the pelvis were considered malignant tumors (Fig. 1). Thyroid B-ultrasound showed no tumor lesions. After exclusion of contraindications to surgery, the patient underwent surgical exploration on July 26, 2022, during which frozen pathological examination was performed. The patient recovered well and was discharged. At the first follow-up visit in October 2022, the patient had an excellent survival.

### 2.1. Pathological examination

Frozen macroscopic examination of the left ovarian tumor showed a gray-yellow nodule, 3.5 × 3.5 × 2.5 cm^3^, and the section was patchy gray white, gray red, and hard, with local bleeding. Microscopic morphology was consistent with well-differentiated cyst-like structures of varying sizes. Eosinophilic secretions were seen in some cysts, and tumor cells were of the same size; the frozen diagnosis of left ovary was struma ovarii, and the biological behavior was considered malignant. After the diagnosis was made, the patient underwent total hysterectomy with double appendectomy, greater omentum resection, and pelvic mass resection.

**Figure 2.**
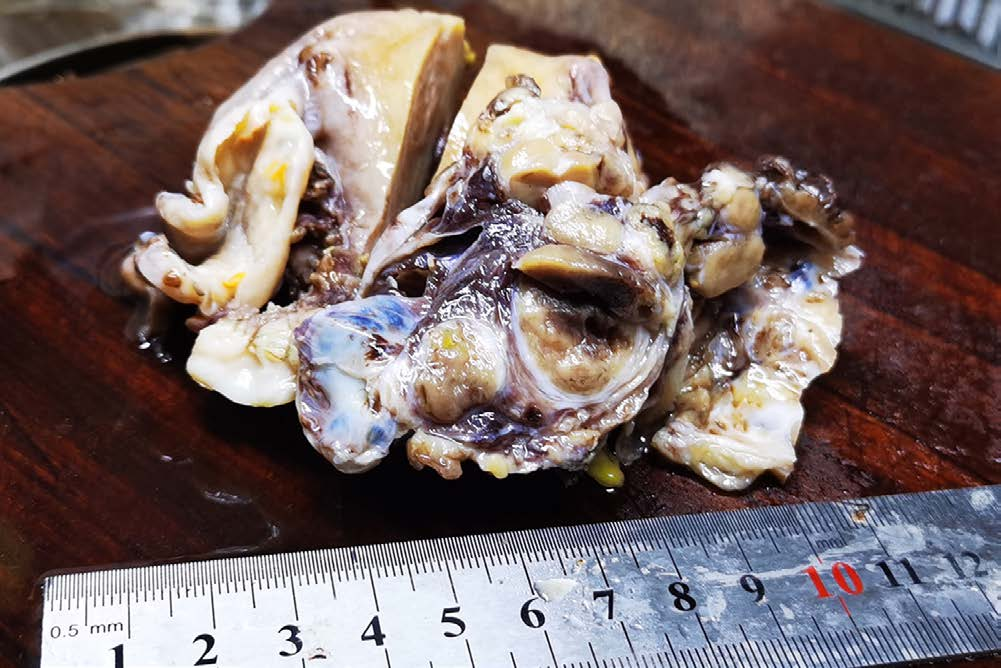
The right ovary is occupied by tumor nodules of different sizes.

**Figure 3.**
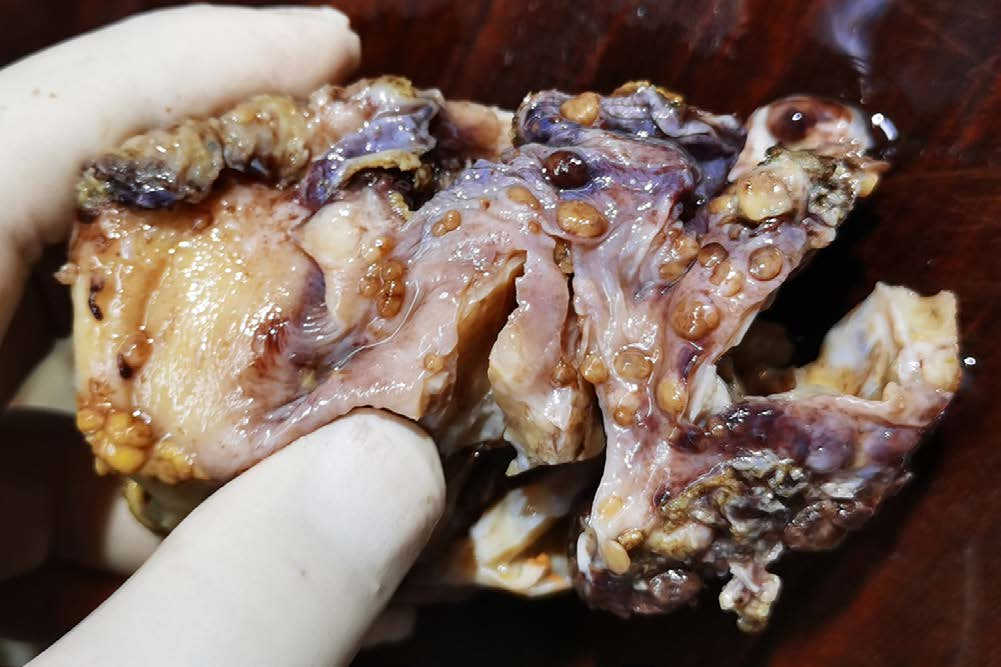
Multiple tumor nodules are seen on the surface of uterine myometrium.

**Figure 4.**
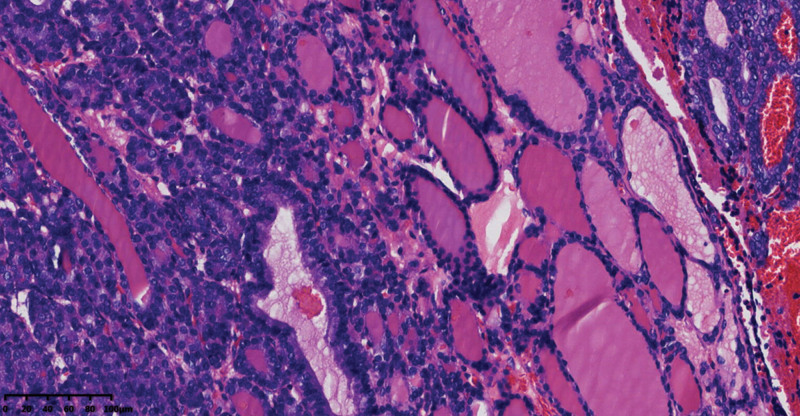
The transition area between benign struma ovarii and follicular carcinoma can be seen in the focal area.

**Figure 5.**
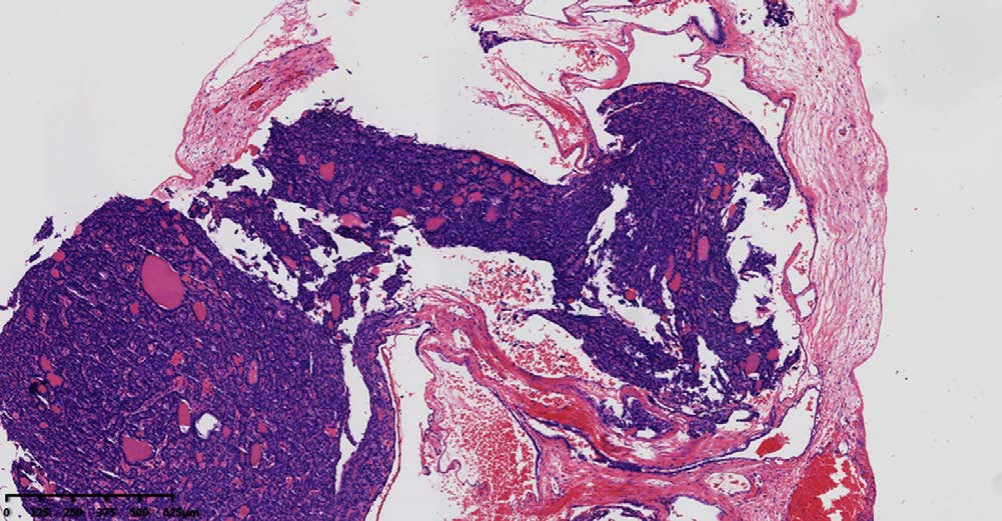
Nodules on the surface of uterus, focal areas show tumor cells growing into blood vessels.

**Figure 6.**
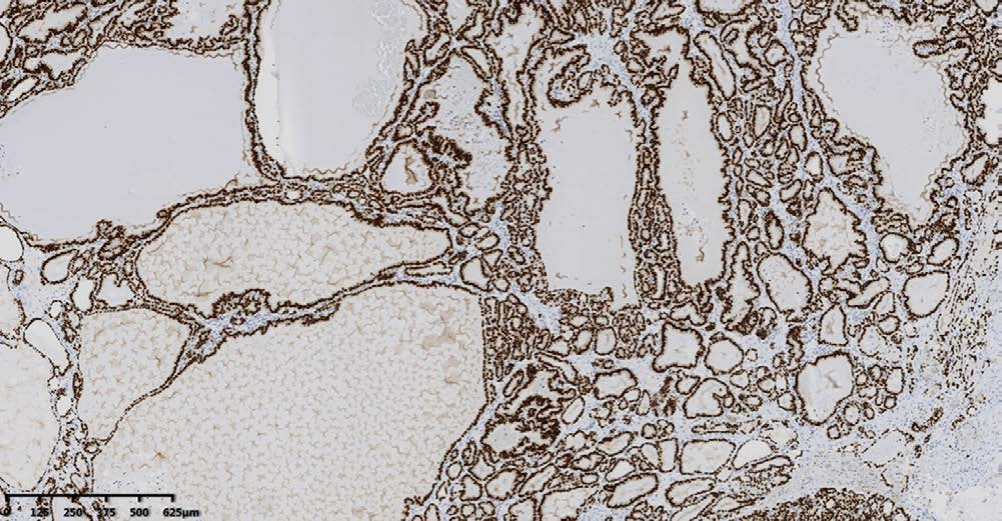
TTF-1 expression is positive. TTF-1 = thyroid transcription factor-1.

Pathological examination of the gross total hysterectomy specimen revealed a size of 9.0 × 6.0 × 3.5 cm^3^, with a smooth cervical surface, with a 0.2 cm thick endometrium, and 2.0 cm thick muscle wall. Multiple dark red miliary nodules were seen on the surface of uterine muscle wall, and the left ovary was 3.0 × 2.0 × 2.0 cm^3^, gray white and soft on the section. A right oval tumor was seen in the right adnexal area, sized about 7.0 × 4.0 × 3.5 cm^3^, and multiple dark red nodules were seen inside the gray red section with bleeding (Fig. 2). Microscopy revealed follicular epithelial cells arranged in nodular shapes of different sizes, infiltrating in the ovarian stroma, with a visible vascular tumor thrombus. The follicular epithelial cells were arranged closely and were slightly heteromorphic, but were not accompanied by papillary carcinoma features such as nuclear sulcus and intranuclear inclusion bodies (Figs. 3, 4, and 5). Proliferating tumors cells were immunohistochemically positive for thyroglobulin, thyroid transcription factor-1, cytokeratin19, CD56 (partially), Proliferation Marker (Ki-67, 5%), and CD34, and was negative for hector battifora mesothelial antigen-1, chromogranin A, synaptophysin, and Inhibin-*α* (Fig. 6). The TTF1 positivity indicated that the tumor had originated from the thyroid epithelium. CD56 positivity ruled out papillary thyroid cancer, and the CD34 positivity indicated vascular invasion of tumor cells. Immunohistochemical results combined with microscopic morphology allowed a diagnosis of follicular carcinoma originating from struma ovarii.

## 3. Discussion

Struma ovarii is a rare, ovarian, single germ teratoma, accounting for 0.5% to 1% of all ovarian tumors and 5% of ovarian teratomas.^[2]^ Struma ovarii must be widely sampled, as some well differentiated thyroid tumors and follicular carcinoma show small tumor cell heterogeneity. In the past, some researchers have called this well-differentiated tumor “malignant ovarian goiter,” but have not clearly described the histological type of the tumor. Therefore, Roth et al proposed the concept of “highly differentiated follicular carcinoma of ovarian origin derived from struma ovarii.” When the typical histological morphology is observed, the potential pathological diagnoses include papillary carcinoma, follicular carcinoma, or other specific diagnostic terms.^[4]^

The onset of follicular carcinoma originating from struma ovarii is insidious, often with lower abdominal and anal discomfort caused by pelvic mass as the first symptom.^[5]^ The unique point of this case is that the patient underwent laparoscopic cystectomy of the right ovarian cyst in the external hospital 2 years prior. At final presentation, the pathological diagnosis was struma ovarii accompanied by active follicular epithelial hyperplasia, with visible focal necrosis. The tumor was large, and its biological behavior was difficult to determine, so further examination was required to determine its nature. No mass was found in the left ovary during operation. However, the patient did not undergo any further examination or treatment as there was no obvious discomfort after the operation. The main diagnostic basis of follicular carcinoma originating from struma ovarii was that the tumor originated from malignant transformation of struma ovarii, and is characterized by infiltration of tumor cells into the ovarian stroma and invasion of blood vessels; accompanied by recidivism of surrounding organs or distant metastasis.^[6]^ The diagnostic criteria of follicular carcinoma confined to the ovary are essentially the same as those of thyroid follicular carcinoma in the neck. Tumor cells have a certain degree of atypia, infiltrate the ovarian stroma and invade the vasculature. These cells differ from those of struma ovarii in that they have a certain degree of atypia, invasive growth in the ovarian stroma, and vascular involvement. Therefore, it was very unfortunate that the patient did not undergo further immunohistochemistry at that time.

During this operation, the surgeon noted several quasi round solid masses in the pelvic cavity on the surface of the uterus, sigmoid colon, greater omentum, peritoneum, and diaphragm. It was unclear whether these extensive lesions were caused by the surgical implants 2 years prior or by extensive infiltration and metastasis caused by the continued growth of residual tumor cells. Roth et al previously proposed 2 explanations for this situation; firstly, that these lesions originate from struma ovarii or mature cystic teratoma with thyroid components folllowing capsule damage or tumor rupture; and secondly, that these lesions originate from the dissemination of well differentiated follicular carcinoma, and may be the manifestation of thyroid follicular carcinoma at a certain stage.^[4]^ As with other ovarian malignancies, these lesions can migrate to the pelvic or abdominal lymph nodes through the lymphatic system, and can be directly implanted in the greater omentum, abdominal cavity, and transferred to the bone, brain, lung, and liver.^[7,8]^ Since follicular carcinoma originating from ovarian goiter and thyroid follicular carcinoma have similar molecular genetic abnormalities, the histological morphology of the 2 is similar. Gene alterations in follicular thyroid carcinoma include point mutations in HRAS, NRAS, and KRAS genes. In ovarii thyroid cancer, NRAS gene mutations may be associated with lymph node and distant metastasis.^[9,10]^

Benign struma ovarii is treated with adnexectomy on the affected side. When complicated with benign implantation, this treatment is supplemented with lesion resection.^[6]^ For follicular carcinoma originating from struma ovarii or those with recurrence, total hysterectomy, and bilateral adnexectomy with resection of metastatic foci can be performed. Patients with recurrence or metastasis may consider total thyroidectomy with Iodine131 adjuvant therapy.^[11]^ In recent years, it has been reported that the struma ovarii can convert to malignant thyroid follicular carcinoma for many years after operation. Therefore, careful exploration should be conducted during the operation of struma ovarii, all samples should undergo pathological examination, and long-term close follow-up should be conducted after the operation. Serum thyroglobulin can be used as a detection index for tumor recurrence after operation.^[12]^

In summary, this case report confirmed that follicular carcinoma originating from struma ovarii is prone to recurrence and metastasis. Furthermore, the analysis of the microscopic morphological characteristics and immunohistochemistry deepened our understanding of the pathological characteristics of ovarian and thyroid follicular carcinoma, and further provides a diagnostic reference for other clinicians who will encounter these conditions in the future. Simultaneously, this experience deepened our understanding of the etiology, diagnosis, differential diagnosis, biological behavior, and prognosis of follicular carcinoma originating from struma ovarii.

## Acknowledgements

The authors thank the patient for their consent for this case report to be published.

## Author contributions

**Conceptualization:** Leyi Shou, Junjie Yang, Yiqian Jiang.

**Methodology:** Jianfeng Lu.

**Resources:** Huabing Wang.

**Visualization:** Lijun Sun, Hao Dong.

**Writing – original draft:** Leyi Shou

**Writing – review & editing:** Yiqian Jiang.
